# Induced Pluripotent Stem Cell-Derived Red Blood Cells and Platelet Concentrates: From Bench to Bedside

**DOI:** 10.3390/cells7010002

**Published:** 2017-12-27

**Authors:** Daniele Focosi, Giovanni Amabile

**Affiliations:** 1 North-Western Tuscany Blood Bank, Pisa University Hospital, via Paradisa 2, 56124 Pisa, Italy; 2 ADIENNE, via Zurigo 46, 6900 Lugano, Switzerland

**Keywords:** iPS cells, transfusion, red blood cells, platelets

## Abstract

Red blood cells and platelets are anucleate blood components indispensable for oxygen delivery and hemostasis, respectively. Derivation of these blood elements from induced pluripotent stem (iPS) cells has the potential to develop blood donor-independent and genetic manipulation-prone products to complement or replace current transfusion banking, also minimizing the risk of alloimmunization. While the production of erythrocytes from iPS cells has challenges to overcome, such as differentiation into adult-type phenotype that functions properly after transfusion, platelet products are qualitatively and quantitatively approaching a clinically-applicable level owing to advances in expandable megakaryocyte (MK) lines, platelet-producing bioreactors, and novel reagents. Guidelines that assure the quality of iPS cells-derived blood products for clinical application represent a novel challenge for regulatory agencies. Considering the minimal risk of tumorigenicity and the expected significant demand of such products, ex vivo production of iPS-derived blood components can pave the way for iPS translation into the clinic.

## 1. Why We Need Alternatives to Donated RBCs and Platelet Concentrates

Red blood cells (RBCs) and platelets (PLT) transfusion are the main prophylactic and therapeutic option in severe anemia and thrombocytopenia, respectively.

This has led the World Health Organization to include blood within the Model List of Essential Medicines, point 11.1 [1] in accordance with the World Health Assembly resolution WHA63.12. While donation has satisfactorily managed major issues in terms of supply and safety, there are still several limitations to take in consideration. Platelet products are stored at room temperature with gentle agitation to best maintain their viability, however they also have a short shelf life of only up to five days based on both their hemostatic capacity and the risk of bacterial contamination. Therefore, the constant restocking of platelet products is required. Furthermore, blood donors are often unreliable due to weather- or holiday-dependent supply shortages, or wasted excess of platelet products.

Importantly, progressive population ageing in westernized countries will likely lead to a reduction in number of blood donors and an increase of blood recipients. In fact, the Finnish transfusion registry data already demonstrated that the 70- to 80-year-old population has an eight-fold higher RBCs consumption compared to 20- to 40-year-old recipients [2].

Both erythroblasts and megakaryocytes (precursors of red blood cells and platelets, respectively) are difficult to expand in vitro. The in vitro differentiation process from hematopoietic stem cells (HSCs) is relatively short [3] and, unfortunately, the HSCs number that can be achieved by donation is pretty low and hardly scalable. As a consequence, the attention has turned to pluripotent stem cells. Importantly, both in pluripotent stem cell derivation and in physiologic hemopoiesis, both RBCs and megakaryocytes are derived from CD235a^+^CD41a^+^ common megakaryocyte-erythroid progenitor (MEP) [4,5]. In 2008 Lu et al. reported differentiation of human embryonic stem cells (hESCs) into functional oxygen-carrying erythrocytes on a large scale (10^10^–10^11^ cells/6-well plate hESCs), with up to 60% enucleation rate [6]. In 2014, Igor Sluvkin’s group at University of Wisconsin reported that GATA2 and TAL1 transcription factors are capable to directly convert hESC to endothelium, which subsequently transforms into blood cells with erythro-megakaryocytic potential. This process resulted to be significantly efficient with generation of 33 million of CD43^+^ cells from one million transduced H1 hESCs after seven days of expansion [7]. Nevertheless, ethical concerns regarding ESCs are still high [8], and this is also the reason of why induced pluripotent stem (iPS) cells currently represent the alternative approach for blood cells and components derivation.

## 2. iPS Cells Technology

iPS cells were generated for the first time from murine fibroblasts by Shinya Yamanaka’s team by using ectopic expression of transcription factors Oct4, Klf4, Sox2, and c-Myc (OKSM) [9]. In 2007, Yamanaka’s and Thomson’s groups successfully reprogrammed primary human fibroblasts to human iPS (hiPS) cells using the OKSM cocktail [10] or Oct4, Klf4, Sox2, and LIN28 [11], respectively.

Due to transformation concerns, many groups later replaced the c-Myc proto-oncogene [12,13] with less dangerous and programmable genes, such as PARP1 [14,15,16,17,18,19]. Since the landmark finding that lineage-restricted cells can be converted to a pluripotent state, several iPS cell lines have been obtained from patients affected by congenital and acquired hematological diseases, including leukemia, with the purpose to establish disease modeling and identify novel therapeutic targets [20,21,22].

However, the direct use of the iPS cells in regenerative medicine is still delayed by concerns regarding their potential tumorigenicity. Specifically, tumorigenicity of undifferentiated iPS cells contaminating the differentiated cell populations is one of the highest barriers to the clinical use of iPS cells. Nucleus-free cell populations, such as red blood cells and platelets, can be eventually purged of nucleated precursors and undifferentiated cells by irradiation or clinically-approved pathogen reduction technologies (PRT) [23,24].

IPs-derived, pathogen-free, autologous or universal blood carries the potential to alleviate or eliminate the allogeneic blood shortages [25]. Small-scale bioreactors with disposable kits currently allow for in-hospital expansion of suspension cell cultures [26]; on an industrial scale, large-scale bioreactors would allow bulk production of iPS cells in desired amounts and potentially with no Hayflick limit. Peripheral blood has always been considered an easier route to obtain raw material for iPS cells generation. Chen et al., in 2014, first reported derivation of transgene integration-free hiPS cells from a single drop (10–20 µL) of finger-prick blood (stored at 4 °C up to 48 h) in 30–45 days (10 to 15 days for cytokine-driven blood cell expansion and 20 to 30 days for reprogramming), with an efficiency of 0.01% (i.e., 1–2 colonies per blood drop) [27].

Since 2007, most iPS cells manufacturing methods initially relied on fibroblasts as source cell, mouse embryonic fibroblast (MEF) as feeder cells and calf serum added at some point during their culture. Since fibroblasts are not that easy to sample from humans, and both MEFs and serum can potentially be contaminated with xenogeneic pathogens, peripheral blood stem cells have superseded fibroblasts as a cell source, and chemically-defined serum-free and xeno-free protocols have been developed for both generation of iPS cells [28] and for re-differentiation of the pluripotent cells into blood cell derivatives [29,30,31].

In vitro differentiation of iPS cells into mature blood cell types, is typically based on a sequential addition of cytokines at defined concentrations [32], and currently represents the most difficult step in blood cells manufactured from iPS cells [32].

## 3. Advances in RBCs Derivation from iPS Cells

The major limitation for translating iPS cells-derived RBCs into the clinic are: (1) inefficient enucleation; (2) difficulty in switching to adult-type (β) globin form; and (3) the possibly insurmountable number of RBCs (10^12^) needed to generate a single transfusion unit.

A number of groups [33,34,35] have reported successful differentiation of iPS cells down the erythroid lineage using a variety of culture systems (stromal feeder-dependent or independent), generating orthochromatic erythroblasts and reticulocytes (up to 10%): Kobari et al. reported an exceptionally high 26% enucleation rate [36]. The differentiated cells in most reports expressed fetal and embryonic globins, indicating reprogramming of the globin locus from the original parental cell. Erythroid differentiation has been confirmed by morphological analysis and expression of a very limited number of RBCs markers, including sialophorin (CD43) [37], glycophorin A, and transferrin receptor [34,35]. The reticulocytes generated from iPS cells exhibit an oxygen binding capacity similar to cord blood RBCs, which contain predominantly fetal hemoglobin [36], however, the dynamics of the gene expression program (largely erased during reprogramming) found during erythropoiesis from hiPS cells was found distinct compared with adult and cord blood progenitors [38]. Nevertheless, the proteome analysis of erythroid cells differentiated from iPS cells lines was found to be 98% similar to that of normal adult erythroid cells [39], and hiPS cells were able to differentiate along various stages of physiological erythropoiesis [40].

The iPS derived- erythroid cells predominantly express ε- and γ- but little levels of β-globin, likely due, to the low level of Erythroid Krüppel-like factor 1 (EKLF/KLF1) and the absence of BCL11A in these cells. Both genes are largely demonstrated to be required for the developmental switch from fetal to adult globin expression [41,42] and the low expression levels might impact the correct development of the in vitro-derived erytroblasts. Paradoxically, human MSC-derived iPS cells sacs (multiple cysts demarcated by monolayers of endothelial-like cells and retaining CD34+ cells) from sickle cell disease (SCD) patients allow for a more efficient erythroid cell generation with higher β-globin production, likely due to heightened emergence of immature progenitors [43].

To activate KLF1 at defined time points during later stages of the differentiation process and to avoid transgene silencing that is commonly observed in differentiating pluripotent stem cells, Yang et al. targeted a tamoxifen-inducible KLF1-ERT2 expression cassette into the AAVS1 locus. Activation of KLF1 at day 10 of the differentiation process when hematopoietic progenitor cells were present, enhanced erythroid commitment and differentiation. Continued culture resulted in the appearance of more enucleated cells when KLF1 was activated [44].

On the other hand, Sivalingam et al. reported a serum-free and chemically-defined microcarrier-based suspension culture platform for scalable hiPS cells expansion and embryoid body (EB) formation. Improved survival and better quality EBs generated with the microcarrier-based method (including primary human mesenchymal stromal cells co-culture) resulted in a significantly improved mesoderm induction and, when combined with hematopoietic differentiation, resulted in at least a six-fold improvement in hematopoietic precursor expansion, potentially culminating in a 80-fold improvement in the yield of RBCs generation compared to a conventional EB-based differentiation method [45]. Similarly, Olivier et al. described a good manufacturing practice (GMP)-compatible, feeder-free and serum-free method to produce large numbers of fetal erythroid cells from hiPS cells, with a single hiPS cell producing up to 50,000–200,000 erythroid cells by day 31 [30].

Nizhizawa et al. demonstrated that hematopoietic commitment of iPS cells to hematopoietic precursors correlates with IGF2 expression level, which in turn depends on signaling-dependent chromatin accessibility at mesodermal genes [46]. Continuous estrogen (E_2_) signaling is required to promote hematopoietic output from hiPS cells. Supplementation of E_2_ or an ER-α selective agonist significantly increased the number of hemangioblasts and hematopoietic progenitors, and subsequent erythropoiesis [47].

We also demonstrated that it is also possible to obtain in vivo differentiation of iPS cells by way of teratoma formation when iPS cells are injected in immunocompromised mice [18]. This approach is based on the finding of an active hematopoiesis occurring during teratoma formation in regions that have been defined bone-marrow-like structures in which all three hematopoietic lineages were identified, including erythroblasts and megakaryocytes [18]. We speculated that the hematopoietic differentiation through teratoma formation could be of high value due to the differentiation conditions that closely recapitulate the physiologic environment and the facility to isolate specific cell types or derivatives through the Fluorescence-Activated Cell Sorting (FACS) approach. The scale-up of this mentioned approach is one of the challenges still open before the clinical application of in vivo-derived blood components from iPS cells.

Giani et al. showed that suppression of SH2B3 (a negative regulator of cytokine signaling for which naturally occurring loss-of-function variants increase RBCs counts in vivo) in primary human HSCs enhanced the maturation and overall yield of in vitro-derived RBCs. Moreover, inactivation of SH2B3 by CRISPR/Cas9 genome editing in human hES cells allowed enhanced erythroid cell expansion preserving differentiation [48]. A similar genetic engineering approach could be used for iPS cells.

Taken together, the several findings obtained by independent research groups suggest that there is still a lack of data addressing the function of iPS cells-derived RBCs (e.g., the capacity to release oxygen to tissues under normoxia and hypoxia) in vivo by using relevant animal species. We believe that this should be the focus of investigation in the near future.

Apart from transfusion applications, understanding RBCs re-differentiation from iPS cells in vitro is also fundamental to recapitulate defects in erythroid differentiation and boost drug discovery [49] or to test the effectiveness of gene-edited iPS cell-derived HSCs [50,51].

## 4. Advances in Platelet Derivation from iPS Cells

Production of pathogen-free, O donor platelet concentrates with negligible isoagglutinin titers and no strong antigen expression is the gold standard in the field. Despite the fact that megakaryocytes can be directly converted from fibroblasts (using NF-E2, MAFG, and MAFK [52], or GATA2, RUNX1, GATA1, TAL1, LMO2, and c-MYC [53]), derivation from iPS cells promises an easier scalability. Platelet function is usually tested by measuring aggregation or adhesion under shear stress, eventually combined with viscoelastic test [54].

In 2010, Takayama et al. successfully produced platelets from individual four-factor hiPS cell clones established from human dermal fibroblasts (HDFs). These human iPS cells-derived platelets showed a satisfactory functionality in vitro and in vivo in NOG (nod-scid/IL-2 γc-null) thrombocytopenia mouse model [55]; however, large scale platelet production was not tested in that study [56]. Similarly, Nishimura et al. generated, for the first time, MKs and functional platelets in vitro using canine induced pluripotent stem cells (ciPS cells) obtaining the 29% ratio of peripheral platelets CD41^+^/61^+^ and 17% of platelets from culture supernatant, which demonstrated the possibility of MKs generation and the release of functional platelets from iPS cells [57].

Little research about large-scale megakaryopoiesis and thrombopoiesis from iPS cells has been conducted so far. Feng et al. generated universal platelets from hiPS cells in the absence of serum and animal feeders in less than 20 days. In total they generated 2 × 10^9^ megakaryocyte progenitors (MKPs) from 1.26 × 10^8^ iPS cells, an average of >16 MKPs per single iPS cells input. Significantly, they deleted the β_2_-microglobulin gene, generating “universal platelets” that are negative for the majority of histocompatibility antigens [29]. The same group also demonstrated that approximately 30 platelets per iPS cells-derived MK could be generated in vitro in the presence of shear force [58]. However, such efficiency can still be considered low compared to >2000 platelets/MK occurring in the human bone marrow. Recently, Nakamura et al. [59] sought to establish stable immortalized megakaryocyte progenitor cell lines (imMKCLs) for clinically-applicable generation of iPS cells-derived platelets. However, the technology of manufacturing functional platelets ex vivo from iPS cells requires sophisticated reprogramming method. Thus, the challenge for reaching industrial scale generation of ex vivo platelets is still present. Simpler culture systems and less strict experiment protocols for platelet generation from iPS cells are needed to take the next step forward.

Megakaryocyte (MK) cell lines with robust proliferation capacity have been established from hiPS cells by the introduction of specified sets of genes, such as GATA2 and TAL1 [31]. These expandable MKs are also cryopreservable and, thus, would be suitable as master cells for good manufacturing practice (GMP)-grade production of platelets, assuring availability on demand and safety against blood-borne infections. Meanwhile, developments in bioreactors that physically mimic the in vivo environment and discovery of substances that promote thrombopoiesis have yielded competent platelets with improved efficiency. The derivation of platelets from iPS cells could further resolve transfusion-related alloimmune complications through the manufacturing of autologous components and human leukocyte antigen (HLA)-compatible platelets from stocked homologous HLA-type iPS cells libraries or by manipulation of HLAs and human platelet antigens (HPAs). Considering these key advances in the field, HLA-deleted platelets could become a universal product that is manufactured at industrial level to safely fulfill almost the entire demand [60]. However, culture at 37 °C induces ectodomain shedding on platelets of glycoprotein Ibα (GPIbα), the von Willebrand factor receptor critical for adhesive function and platelet lifetime in vivo, through temperature-dependent activation of a disintegrin and metalloproteinase 17 (ADAM17). The selective ADAM17 inhibitor, KP-457 effectively suppresses the shedding, leading to the production of functional human iPS cells-derived platelets at 37 °C [61].

Borger et al. silenced the expression of HLA class I to generate a stable HLA-universal iPS cells line that can be used as a renewable cell source for the generation of low immunogenic cell products. The expression of HLA class I was silenced by up to 82% and remained stable during iPS cells cultivation. In that study, the authors focused on the generation of megakaryocytes (MK) and PLTs from a HLA-universal iPS cells source under feeder-free and xeno-free conditions. On day 19, differentiation rates of MKs and PLTs with means of 58% and 76% were observed, respectively. HLA-universal iPS cells-derived MKs showed polyploidy with DNA contents higher than 4n and formed proPLTs. Importantly, differentiated MKs remained silenced for HLA class I expression. HLA-universal MKs produced functional PLTs. Notably, iPS cells-derived HLA-universal MKs were capable to escape antibody-mediated complement- and cellular-dependent cytotoxicity [62].

Feng et al. recently reported a serum and animal feeder-free method that permits differentiation of hiPS cells into MKs and functional platelets in less than 20 days and cryopreservation of MK progenitors, enabling a rapid “surge” capacity when large numbers of platelets are needed. IPS cells-derived platelets form aggregates, lamellipodia, and filopodia after activation and circulate in macrophage-depleted animals and incorporate into developing mouse thrombi in a manner identical to human blood platelets. By knocking out the β_2_-microglobulin gene, platelets that were negative for class I HLA have also been generated [29,63].

Kammers et al. studied genetic and transcript integrity of iPS cells-derived MKs detecting a very low rate of genotype discordance with parent cell (0.0001–0.01%), no CNVs, and highly biologically-relevant gene expression profiles [64].

Moreau et al. reported large-scale generation of MKs in chemically-defined conditions (a two-day exposure to FGF2, BMP4, and LY-294002, followed by a further 10 day culture in medium containing TPO and IL1β) using a forward programming strategy relying on the concurrent exogenous expression of 3 transcription factors: GATA1, FLI1, and TAL1. The forward programmed MKs proliferate and differentiate in culture for several months, with MK purity over 90%, reaching up to 2 × 10^5^ mature MKs per input hiPS cell. Functional platelets were generated throughout the culture allowing the prospective collection of several transfusion units from as few as 1 million starting hiPS cells. The high cell purity and yield achieved by MK forward programming, combined with efficient cryopreservation and good manufacturing practice (GMP)-compatible culture, make this approach eminently suitable to in vitro production of platelets for transfusion [65]. An important step forward in the clinical use of iPS cells-derived blood components will definitively be the expansion though bioreactors. To this aim several systems have been recently proposed [60].

While bottlenecks in low megakaryocyte ploidy and a low amount of shed platelets remains, Advanced Cell Technology, Inc. and Megakarion (a spin-off the Kyoto University’s Center for iPS Cell Research (CiRA)) are currently investing on clinical trials.

## 5. Conclusions

RBCs and platelets represent ideal candidates for clinical trials based on iPS cells-differentiated cell therapies. The proteome analysis of these blood components precursors differentiated from iPS cells lines has been found to be very similar to the one of normal adult blood cells raising enthusiasm in the hematology community.

**Figure 1:**
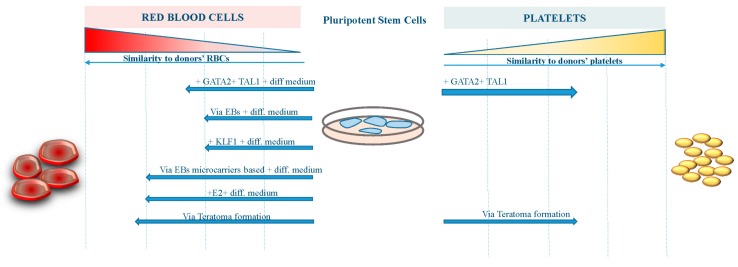
Focosi D and Amabile G.

However, further work to improve the differentiation procedures and the yield of erythroid cells from existing iPS cell lines is required before iPS cell-derived RBCs become suitable for transfusion therapy (Figure 1). On the other hand, platelets derived from iPS cells are now approaching the clinical stage, having achieved a reasonable amount of functionality and manufacturing milestones (Figure 1).

Engineering technology, such as bioreactor use, will also be necessary for successful advances, but details and costs still remain the main hurdles. If these hurdles will be overcome, then we will end up with several advantages in terms of ease of availability and costs to the health system [66], opening a new perspective for patients affected by severe underserved hematological conditions.

The figure shows the grade of similarity reached by RBCs and platelets derived from pluripotent stem cells with different approaches compared to human bone marrow-derived blood components.

## Supplementary Materials

Supplementary File 1

## Abbreviations

iPS cells	Induced pluripotent stem cells
RBCs	Red blood cells
PLT	Platelets
