# Supplementary figures and images for: Induced Pluripotent Stem Cell-Derived Red Blood Cells and Platelet Concentrates: From Bench to Bedside
Source: Cells. 2017 Dec 27;7(1):2. doi: 10.3390/cells7010002 (PMC5789275; doi:10.3390/cells7010002)

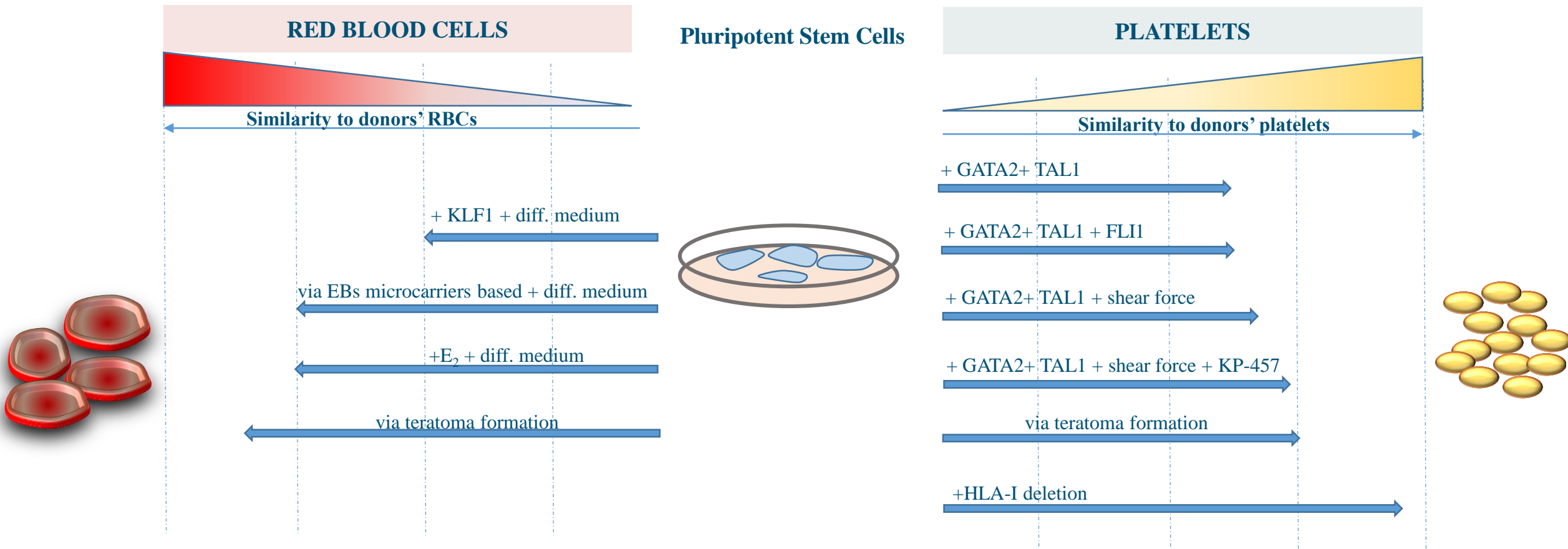

Figure 1. Focosi D. and Amabile G.

Supplement: Supplementary File 1 [file cells-07-00002-s001.pdf]
